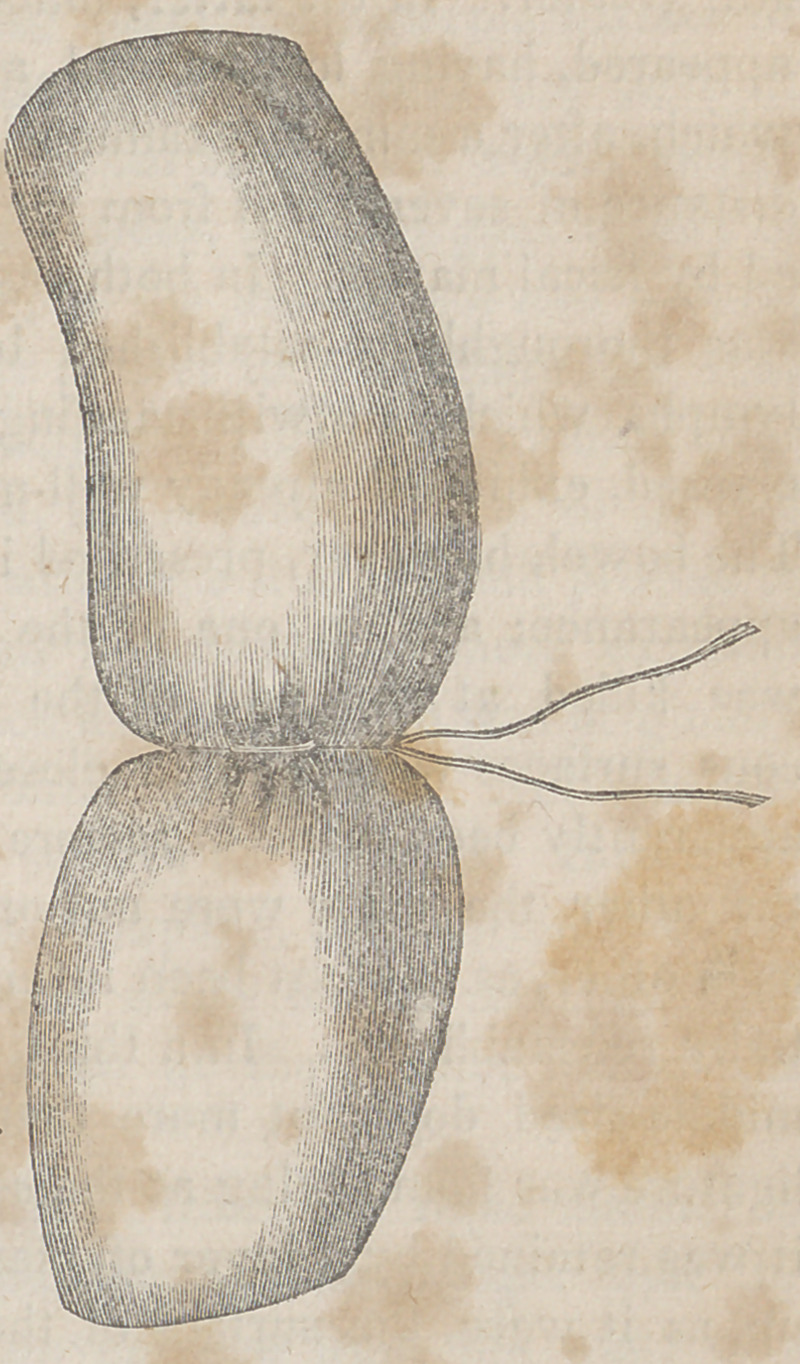# An Experimental and Critical Inquiry into the Nature and Treatment of Wounds of the Intestines

**Published:** 1843-01

**Authors:** Samuel D. Gross

**Affiliations:** Professor of Surgery in the Louisville Medical Institute


### THE WESTERN JOURNAL OF MEDICINE AND SURGERY.

---

JANUARY, 1843.

---

Art. I.—*An Experimental and Critical Inquiry into the Nature and Treatment of Wounds of the Intestines.* By Samuel D. Gross, M. D., Professor of Surgery in the Louisville Medical Institute.

A monograph on wounds of the intestines has been an acknowledged desideratum with the profession. The work of Mr. Travers, the only production of the kind in the English language, has been out of print nearly a quarter of a century, and hence the only information accessible to practitioners, especially those of the United States, is such as is to be found in the various periodicals of the day, or in our systematic treatises on surgery. The latter, unfortunately, contain little, if any thing, that is worthy of reliance; they enter into no details, and some of them do not even allude to the subject; a circumstance so much the more surprising when we reflect upon the importance of these injuries, and the attention which has been bestowed upon them by some of the most respectable members of the profession. In the present undertaking an attempt has been made to supply this deficiency, by exhibiting a connected view of the subject, embracing an account of the results of my own researches, and of those who have preceded me in the same field of inquiry.

My investigations were commenced in the spring of 1841, and continued, with various intermissions, until a few months ago. The object was, in the first place, to inquire into the process employed by nature in repairing wounds of the intestinal tube; and secondly, and more particularly, to determine, if possible, the value of the various methods of treatment that have been recommended from the time of Ramdohr down to the present day. The experiments, upwards of seventy in number, were performed exclusively on the dog, as the most eligible animal that could be procured for the purpose, with the assistance of my private pupils, Messrs. Wendel, Comstock, Baker, Shumard, Church, Grant and Williams. Many of them were also witnessed by Mr., now Dr. Hagan, by Dr. Colescott, one of the Editors of the Western Journal of Medicine and Surgery, by Mr. Mullen, and by Dr. Richard Ferguson, of this city. To the latter gentleman, who has kindly furnished most of the accompanying drawings, I am desirous thus publicly to tender my acknowledgments.

It is proper to state that an abstract of this essay was read before the Medical Convention of Ohio, at its last meeting at Cincinnati, in May 1842.

The notions of the older writers respecting the nature and treatment of wounds of the intestines were, for the most part, exceedingly crude and erroneous. Neglecting to institute experiments for their successful elucidation, they contented themselves with such facts as they witnessed in the human subject; and as these were not only few, but generally imperfectly noted, the conclusions which they deduced from them were far from throwing any real and substantial light upon this interesting branch of pathology. Indeed, until the publication of the researches of Mr. Travers, of London, early in the present century, the management of wounds and injuries of the alimentary tube was altogether empirical, being regulated rather by accident than sound principles derived from the study of healthy and morbid action. His labors in this department, conducted as they were at an early period of his professional life, evinced no ordinary judgment and talent, and served as a happy presage of the reputation which has since awaited him. They are comprised in an octavo volume of nearly four hundred pages, including a complete summary of all that was known on the subject at the time at which it appeared in 1812. It is entitled: "*An Inquiry into the Process of Nature in Repairing Injuries of the Intestines; Illustrating the Treatment of Penetrating wounds and Strangulated Hernia;*" and is one of the most able and philosophical productions that have enriched the science of surgery within the last fifty years, so prolific in discovery and improvement.

In the investigations just referred to, Mr. Travers did not, like his predecessors, limit his inquiries to the human subject, but extended them to the inferior animals, upon which, especially the dog, he performed a series of the most interesting experiments, equal in point of beauty and importance to those instituted by his countryman, Dr. Jones, to ascertain the process employed by nature in suppressing hemorrhage from divided arteries. The results of these researches are well-known to the profession, and any further notice of them, excepting in an incidental manner, will therefore be unnecessary in a work of this kind, which is intended more particularly as a record of my own observations and of the facts that have been disclosed within the last quarter of a century. It is but just to say that several years before Mr. Travers issued his work, Dr. Thomas Smith, of the Island of St. Croix, was engaged in making some researches on the same subject, an account of which was published in his Inaugural Dissertation, presented to the Trustees and Faculty of the University of Pennsylvania, in 1805. His object seems to have been rather to inquire into the propriety of using certain kinds of sutures, recommended by Le Dran, Ramdohr, John Bell, and other surgeons, than to ascertain the process employed by nature in effecting reparation. His experiments, twelve in number, were conducted with considerable care, though he has failed, in almost every instance, to notice with proper minuteness the results of his dissections; a circumstance so much the less surprising when we reflect upon the low state of pathological science at the period at which he wrote. Limited as these researches are, and imperfectly as they have been detailed by their author, they nevertheless tended to establish some important practical precepts, to which allusion will be made in another part of this inquiry.

Before I proceed to detail the results of my own experiments, and the inferences which I have been led to deduce from them, it will not be amiss to make some remarks on the structure of the alimentary tube, the arrangement of the peritoneal cavity, and the phenomena which characterize the presence of wounds in the situation in question.

### I.—*Structure of the Alimentary Canal.*

Into the consideration of the structure of the intestinal canal I do not deem it necessary to enter at any length, as it must be familiar to all who have any pretensions to correct anatomical knowledge. It will be sufficient for the object I have in view to make a few remarks respecting the different tunics, and the manner in which they are united to each other.

The *outer membrane* of the intestinal tube belongs to the class of serous textures, and deserves to be mentioned here chiefly on account of the facility with which it takes on inflammation, and the important part it plays in the reparation of traumatic lesions. It is intimately connected, except along the line of reflection of the mesentery and omentum, to the subjacent muscular tunic, by short, dense cellular substance, and consists every where of a single lamella, the strength of which varies in proportion to the age of the individual. In young animals it is easily lacerated, and incapable of withstanding much traction or pressure. Hence, if, in sewing up a wounded bowel, the ligature be carried merely through the serous investment, it will be almost certain to be torn out in the efforts which are necessary to replace the part within the abdomen. When inflamed, this tunic promptly pours out plastic lymph, which, under favorable circumstances, becomes readily organized. If the morbid action runs high, the lymph is generally intermixed with serum, and sometimes even with blood. Pus is a more common attendant on the chronic form of the disease; it is, however, occasionally observed in the acute stage, and that, too, within a very short time after the development of the disorder.

The *muscular tunic,* interposed between the preceding and the cellulo-fibrous, to both of which it is intimately connected, is composed of two planes of fibres, a superficial and deep-seated. The first, which is much the more delicate of the two, is made up of thin, pale fibres, which are arranged longitudinally, and which exhibit certain, but as far as the present inquiry is concerned, unimportant peculiarities in different parts of the tube. The second layer consists of circular fibres, much more distinctly marked than the preceding, which extend in parallel lines round the entire circumference of the bowel, their extremities being inserted as it were into each other.

Lying beneath this muscular plane is the celebrated *nervous tunic*, as it was called by the ancient writers. Alternately admitted by some and rejected by others, this layer has been recently described by Mons. Cruveilhier,* under the name of the *fibrous lamella,* in consideration of its structure, which closely assimilates itself to that class of tissues. It is intimately connected, on the one hand, with the mucous membrane, and, on the other, with the muscular tunic, into the latter of which it sends a large number of processes, of a dense, firm character, which thus tend to strengthen the union between them. In its thickness and consistence it varies in different portions of the canal, being at its minimum in the ileum and colon, and at its maximum in the remainder of the small and large bowels. Strong and resisting, it is semi-transparent, devoid of elasticity, and composed of condensed cellular tissue, in which it is impossible to distinguish any of that linear disposition so conspicuous in the fibrous membranes, properly so called. The filaments of which it consists interlace with each other in every conceivable manner, forming thus a very close net-work, which it is difficult to unravel by

*Anatomie Descriptive, T. ii, p. 470.

insufflation and other artificial processes. In great obesity small particles of fat are occasionally to be seen in its meshes, which always disappear in emaciation, however induced. In chronic affections, especially in such as are of a malignant nature, this tunic is often remarkably altered in its structure, being rendered much thicker than in the normal state, at the same time that it assumes a dense and almost gristly hardness. It readily re-unites when divided, as I have witnessed in numerous experiments, and deserves to be attentively studied, as it is the membrane through which the surgeon should always carry his needle in sewing up wounds of the intestines.

This tunic—for so indeed it should be considered—is much more distinct in carnivorous animals than in herbivorous, or than in the human subject. In the small bowel of the African lion it is an exceedingly firm, dense, and elastic texture, of a white opake aspect, capable of great resistance, and nearly half a line in thickness. In the bear its characters are nearly similar. In the dog it is less strong, and also less distinctly fibrous, yet more so, considerably, than in the human subject. In the horse it forms a thick inelastic layer, of a dull greyish color, which frequently contains a good deal of adeps. In the ox its properties are very much of the same nature.

The *internal membrane*, of a mucous character, varies in thickness and consistence, as well as in the mode of its arrangement, in different parts of the tube, and does not require any particular notice in relation to the subject under consideration. It is sufficient to observe that it is highly vascular and sensitive; that it re-unites with great difficulty, comparatively speaking, when divided; and that, although extremely prone to inflammation, it rarely, when thus affected, deposits plastic lymph, the constant and invariable product of peritonitis.

### II.—*Nature and Extent of the Peritoneal Cavity.*

Is there any cavity, properly so called, in the peritoneal sac, and, if so, what is its extent or capacity? Concerning this question various views have been expressed by anatomists and surgeons, and it is important, therefore, that it should be carefully examined before we pass an opinion on it, either affirmatively or negatively, as our decision, whatever it may be, must be calculated to exert no inconsiderable influence upon the treatment of traumatic lesions of the alimentary canal. Mr. John Bell, in his Principles of Surgery,* affirms that, "there is not, truly, any cavity in the human body, but that all the hollow bowels are filled with their contents—all the cavities with their hollow bowels—and that the whole are equally and fairly pressed." That this is really so every one will admit; but when he declares, as he does almost in the same sentence, that all the viscera of the abdomen may be deeply wounded, and yet no blood or fæces can escape, he makes an assertion which is unsustained by facts, and which daily observations on the human subject, as well as experiments upon the inferior animals, wholly disprove. Examples of fæcal effusion, either alone or in combination with blood, are mentioned by a great number of pathologists, by Hoyerus, Ravaton, and Morgagni, of the last century; by Cooper, Travers, and others, of the present. Indeed, there is literally no end to cases of this description—a volume would scarcely suffice to record them all; for there is hardly a physician, at all extensively engaged in practice, who has not met with them. A few years ago I assisted my colleague, Professor Cobb, in examining the body of a stout, athletic man, who had been stabbed in the abdomen, apparently with a dirk, which had entered near the umbilicus, and perforated the jejunum, laying open that tube in an oblique direction to the extent of nearly half an inch. Through this

*Vol. i, p. 487. London, 1827.

aperture a small quantity of stercoraceous matter had made its way into the peritoneal sac, where it induced violent inflammation, of which the patient died in less than two days.

Moreover, certain pathological facts clearly show the fallacy of the above opinion. In ulceration of the bowels the morbid action occasionally extends to the serous investment, which it at length perforates, leading thus to a discharge of fæcal matter. Of this not less than five or six well-marked cases have fallen under my own observation, and numerous others of a similar kind are narrated by authors. This occurrence must, in fact, inevitably happen whenever nature fails to effect adhesion in the surrounding parts, however slight the opening. In several of my cases the aperture did not exceed two lines, or the sixth of an inch in diameter, and in some of those that have come under the notice of other observers, it was still smaller, scarcely equalling the size of a crow-quill. Additional facts have been furnished by Smith and Travers, in their experiments on dogs. My own researches have afforded the following results.

Having opened the cavity of the abdomen of a small slut, a transverse wound, half an inch long, was made into the jejunum, and the part returned without suture. The animal became sick soon after the operation, and evinced a disinclination to move about. In thirty-two hours she died. The aperture in the bowel was perfectly patulous, with the mucous coat everted, of an oval form, and without the slightest attempt at reparation or adhesion to the circumjacent structures. About six ounces of a dirty yellowish looking fluid, evidently of a fæculent nature, were contained in the peritoneal sac; and there was extensive inflammation of the omentum, together with the serous coat of the bowels, several coils of which adhered with tolerable firmness to each other.

In another experiment, the subject of which was a small dog, and in which the incision was of the same extent and direction, the results were of a similar character. The animal became sick shortly after the operation, and continued in that condition for thirty-six hours, when he died. On dissection, the edges of the wound were found to be in a gaping state, without any apparent effort at restoration; some hardened and fluid fæces had escaped into the abdominal cavity; the bowel was red and contracted for several inches above and below the affected part; and the neighboring knuckles of intestine were agglutinated by plastic lymph.

In a third experiment, in which the wound was only four lines, or the third of an inch in length, and in which the bowel was replaced without suture, recovery occurred without any untoward symptoms, and without any apparent inconvenience to the animal.

Oblique wounds, six lines long, and treated without suture, were followed by the same result as transverse wounds of the same extent. Only two experiments were performed to elucidate this point. The particulars it is unnecessary to detail. It will suffice to say that, in one of the dogs, death took place in thirty-six, in the other, in forty-seven hours, from peritoneal inflammation, occasioned by the effusion of fæculent matter. The wounds in both were in a gaping, patulous state, without any evidence whatever of reparation by the adhesive process.

To ascertain whether a longitudinal wound, six lines long, would be attended with the same degree of danger, was the object of the next experiment. For this purpose a healthy, full grown dog, of moderate size, was selected. Soon after the operation he vomited, and appeared to be in great agony; in thirty-six hours he died. On opening the belly, a considerable quantity of gas, of a highly offensive odor, escaped with a loud noise. Both hardened and fluid fæces were contained in the peritoneal sac, the enteric portion of which, especially in the immediate vicinity of the wound, exhibited marks of violent inflammation. The edges of the wound were separated to the extent of at least two lines, and through the opening thus formed the mucous membrane projected beyond the level of the serous covering. No attempt had been made to re-establish the continuity of the tube by adhesions of the gut to the surrounding parts.

In a second experiment, in which the wound was only four lines long, speedy recovery followed. The dog was a good deal indisposed for the first forty-eight hours, after which he became well and lively, and continued thus until he was killed on the fifteenth day after the operation. A process of omentum occupied the outer wound, which was nearly healed, the small bowels were extensively matted together, and the reparation of the enteric breach had evidently been effected by the adhesion of its edges to the two neighboring coils of intestine. The bottom of the wound was nearly two lines in width at its middle, and imperfectly filled with lymph.

A large dog, killed nine days after having been stabbed with the sword of a cane, two lines in diameter, presented the following appearances: two punctures, distant about five inches from each other, were found in the small bowel; the edges of each were in close contact, and their outer surface was completely covered with plastic lymph, which was quite firm, slightly ecchymosed, and vascular. The animal retained his original embonpoint, and did not appear to have suffered materially from the injury which had been inflicted upon him.

From the foregoing observations and experiments, the following conclusions may be established:

First—that, although there is not, in the true sense of the term, any peritoneal cavity, yet the arrangement existing between it and the enclosed viscera is of such a nature as to admit, and that very frequently, too, with great readiness, the effusion of stercoraceous matter in wounds and ulcerative perforation of the bowels.

Secondly—that wounds of the bowels to the extent of six lines, whether transverse, oblique, or longitudinal, are almost always, if not invariably, followed by the escape of fæcal matter, and the consequent development of fatal peritonitis.

Thirdly—that wounds not exceeding four lines in length, no matter what may be their direction, are not near so apt, if left to themselves, to be succeeded by the extravasation of the contents of the intestinal tube; and that, in the majority of cases, nature, properly aided by art, is fully competent to effect reparation.

These deductions derive additional support from the following experiments, instituted with a view to ascertain the effects of wounds and punctures of different forms and dimensions: 1. A longitudinal incision, two lines and a half in length, immediately contracted to one line and three-quarters, with a sufficient amount of eversion of the mucous lining to close the resultant orifice. 2. A similar wound, four lines long, diminished in a few seconds to three lines, by one line and a half in width; it assumed an oval shape, and the internal membrane protruded on a level with the peritoneal covering, leaving no perceptible aperture. 3. An oblique cut, seven lines in length, contracted to five, by two and a half in width, with marked eversion of the mucous lining. 4. A transverse wound, two lines and a half long, was reduced almost instantaneously to two lines in diameter: it was of a rounded form, and the two outer tunics of the gut retracted so as to expose the mucous membrane. 5. In another experiment, in which the incision, likewise transverse, was half an inch in extent, the orifice assumed a rounded, oval shape, and was reduced to four lines, by two and a half in width, the internal coat exhibiting, as in the other cases, a pouting, or everted arrangement.

These observations are interesting chiefly as showing the efforts which nature institutes to close a breach of this kind, the very moment almost it is inflicted. It is doubtless by a process of this description that the effusion of stercoraceous matter into the peritoneal sac is so generally prevented in those cases in which the solution of continuity is of small extent, not exceeding, for example, a few lines in diameter, and where, consequently, it amounts rather to a puncture than a wound. The eversion of the lining membrane forms a striking and constant feature in injuries of this character, and may be compared, in its effects, to the contraction and retraction observed in the extremities of a divided artery.

It is a circumstance in the highest degree interesting, and worthy of notice, that the eversion of the lining membrane, which is so conspicuous in traumatic lesions of the alimentary tube, is never witnessed in the openings which result from ulcerative action. In the latter, the perforation proceeds in a slow and gradual manner, at the expense mainly of the mucous and fibrous lamellæ, which are always destroyed to a much larger extent than either the muscular or peritoneal. Hence, by the time the ulcer reaches the surface, it is impossible for the lining membrane to protrude across it, as it does when the bowel is wounded by a sharp instrument, a blow, or a kick. Another circumstance which no doubt contributes to produce this result, is the indurated condition of the serous and muscular layers immediately around the perforation, caused by the deposition of lymph during the progress of the ulcerative action.

There is thus a striking difference, as respects their immediate effects, between an opening of the bowel from ulceration and one produced by an incised or lacerated wound. In the former, although it may not be two lines in diameter, extravasation would be almost certain; in the latter, it might be nearly double that size, and yet, for the reason just mentioned, that event, so much to be dreaded, would be little likely to occur.

It is much to be regretted that Mr. Travers, in the experiments which he instituted to illustrate this branch of the subject, as well as in the cases which he has adduced from his own and the practice of others, has not specified the size of the lesion; a matter of such paramount importance that it is only surprising how it could have been overlooked. His chief object, however, appears to have been, not so much to deduce from them any practical precept in reference to the management of such accidents, as to show that the apprehension of intestinal effusion in penetrating wounds of the abdomen, is, in the majority of cases, without foundation. How far he has succeeded in accomplishing this end, I leave it to others to determine.

### III.—*Symptoms, Diagnosis, and Prognosis.*

The next topic into which I proposed to inquire is the consideration of the symptoms of wounds of the intestines. A few remarks under this head will be sufficient for the object in view.

The symptoms of a wounded bowel necessarily divide themselves into two classes, into those, namely, which are furnished by the system at large, and those which are peculiar to the part more directly and immediately implicated. In regard to the first, they are such, generally, as denote a severe shock of the nervous system, but as they are common to this and other injuries, they are of little consequence in enabling us to make out the diagnosis. In almost all instances there is nausea, either alone, or accompanied with vomiting; these symptoms often make their appearance within a few minutes after the infliction of the wound, and continue with great obstinacy for several successive days, or, in fatal cases, until death relieves the patient of his suffering. They are commonly more violent and distressing in lesions of the small than of the large bowel, owing to the more delicate organization of the former than of the latter, and to its more intimate connexion with the stomach and the sympathetic nerves. The prostration of the vital powers is not always in proportion to the extent of the wound, or the danger of the case. Some persons, it is well-known, suffer much more severely from a slight than others do from a violent injury, for reasons which cannot always be explained, but which may be supposed, generally, to be dependent upon some constitutional peculiarity. Reaction is often postponed for ten or fifteen hours after the occurrence of the accident, and until it is fairly established there is sometimes a constant tendency to syncope, with an alarmed and agitated state of the mind, which it is almost impossible to calm or subdue. The countenance under such circumstances has a pale, anxious, and haggard expression; the pulse is small, frequent, and tremulous; the skin is bathed with clammy perspiration; the extremities are cold; the patient tosses about in his bed; the thirst is urgent, as is also the desire for cool air; there are griping pains in the abdomen; and occasionally the discharges from the bowels are involuntary. Conjoined with these symptoms there is sometimes slight delirium with partial blindness or indistinctness of vision.

The *local symptoms* of a wounded intestine are often as equivocal as those which are furnished by the constitution. This must, indeed, always be the case when there is no protrusion of the tube, or when the external opening is so small, or its direction and situation are such, as to prevent effectually the escape of fæces or other matters. It not unfrequently happens that an instrument enters the abdomen, and passes out at the opposite side, directly in the course of the bowels, without in any wise interfering with them. Many interesting and instructive cases of this kind are recorded by writers on military surgery, as well as by civil practitioners, and several will be quoted hereafter in illustration of this part of the subject. The most characteristic signs of this lesion are, unquestionably, the escape of fæces, bile, food or fœtid air from the external wound, and the sudden development of tympanites. The latter symptom, which does not appear to have been sufficiently insisted upon by systematic writers, as very few, if any, mention it, is often present when the others are absent, and may therefore be regarded as in some degree pathognomonic. Jobert thinks it is the most valuable and positive sign of a wound of the intestine that we can have when there is no external opening, or only so small a one as not to permit the egress of stercoraceous or other substances. He relates several instances from his own practice and that of others, in which, by this phenomenon alone, the diagnosis was clearly established. The tympanites supervenes at various periods, from a few minutes to several hours, after the occurrence of the accident, and is always attended with a hollow, drumlike sound on percussion, with tenderness on pressure, and difficulty of respiration. The following cases will more fully explain the nature and importance of this symptom.

A young man, eighteen years of age, of an excellent constitution, an apprentice in a drug-store, in a rencounter with a robber, in May, 1842, was stabbed with a knife in the right side of the abdomen, the instrument entering the anterior wall of the ascending portion of the colon in a transverse direction, and about two inches above the ileo-cœcal valve. The outer wound was fifteen lines in length, and the inner was sufficiently large to allow the escape of a considerable quantity of fæcal matter. A short time after the occurrence of the accident there was diffuse pain of the abdomen, with a discharge of blood from the anus, and at the end of twenty-four hours decided tympanites. The distention progressively augmented for four days, when it had attained an enormous height. From this period it slowly subsided, but did not entirely disappear under a month. Pressure on the abdomen during the first week occasioned the most exquisite pain. The patient finally recovered under the judicious management of Dr. E. S. Williams and Professor Mussey, of Cincinnati, where the case occurred, and where, through the politeness of those gentlemen, an opportunity was afforded me of seeing it, during a visit which I made to that city last summer.

A carriage-driver, sixty years of age, was kicked by a horse upon an old rupture in the left groin, for which he was carried to the St. Louis hospital of Paris. The following morning he had great pain in the belly, especially on pressure, and the swelling, which was very large and emitted a peculiar gurgling noise, was tympanitic. He died the next day under all the symptoms of gangrene or rupture of the intestine. The scrotum, hernia, and belly, were all distended with gas, which could be readily forced from one to the other; the intestinal folds were agglutinated by plastic lymph; black matter was effused into the pelvic and abdominal cavities; and the small bowel was entirely torn across.*

A young man, twenty-one years of age, was thrown from his carriage, the wheel of which passed over his belly. When brought to the St. Louis hospital, immediately after the accident, the skin of the abdomen was found to be perfectly natural, but he complained of great pain, and there was enormous tympanites, the parts on percussion sounding like a drum. His sufferings for eight days were very violent, after which they gradually subsided, and he was rapidly convalescing from his injury, when, at the end of a month, an unexpected attack of pleuro-pneumonitis occurred, which quickly destroyed him. The jejunum adhered to the last false rib, and presented the remains of an opening, which had been completely closed by a sort of plug of the omentum.†

A man affected with cancer of the rectum was admitted into the surgical ward of the St. Louis Hospital under the care of Mons. Richerand. The abdomen became suddenly tympanitic, and this distinguished surgeon at once pronounced the case to be one of intestinal perforation. The autopsy justified the diagnosis. The bowel was found to have given way above the seat of the disease, and thus permitted the escape of the gas upon which the distention depended.‡ Examples of a similar character are recorded by Scarpa, Sévestre, Kapeler, Marjolin, and other writers.

Tympanites, however, does not attend all traumatic injuries of the intestinal canal. When the opening is very small, amounting rather to a puncture than a wound, the escape of gas will either be entirely prevented, or occur only in a small degree, owing to the protrusion of the mucous membrane, which, as was seen in a previous part of this essay, is a constant phenomenon in lesions of this description. A sort of valve

---

*Jobert, Maladies du Canal Intestinal, T. i, p. 61.

†Jobert, op. cit. T. i, p. 62.

‡Jobert, op. cit. T. i, p. 63.

is thus formed, which opposes an effectual barrier to the egress of fæcal matter, intestinal secretions, and even air.

A discharge of blood from the anus is another symptom which, in connexion with some of those already pointed out, is of considerable importance in the discrimination of the lesion before us. Still, as it may, and often does attend other injuries, it cannot be regarded as at all characteristic. The quantity of blood evacuated occasionally amounts to many ounces. In the case previously adverted to, which I saw along with Professer Mussey and Dr. Williams, at least two pints of fluid and grumous blood were discharged during the first three days. It began to pass off seven hours after the occurrence of the injury, nearly unmixed with fæces, and comparatively fresh in its appearance. What was subsequently evacuated was of a darker color, and more firmly coagulated, as if it had been retained for sometime in the bowel.

Equally equivocal is hematemesis, or vomiting of blood which may be enumerated as another, though by no means constant symptom of traumatic lesion of the alimentary tube.

The degree of pain accompanying cases of this kind varies remarkably in different individuals, being very slight in some, and exceedingly severe in others. In most instances it is of a colicky character, though occasionally it is dull and aching, and it is almost constantly increased by pressure, by coughing, and by a full inspiration, especially if some hours have elapsed since the infliction of the injury.

The wound is occasionally complicated with hemorrhage into the peritoneal sac, caused by lesion of the epigastric or internal mammary artery, of some of the branches of the mesentery or omentum, of the aorta or vena cava or of some of their immediate offsets. Unless the abdominal wound be large, very little blood, if any, will appear externally, but it will flow into the serous cavity, where it will occupy the intervals between the intestinal convolutions, descend into the pelvis, or be extensively diffused among the viscera. The amount and rapidity of the effusion will vary in proportion to the size of the wound and the volume of the vessel concerned. When the vessel is very large and the opening considerable, the hemorrhage may be instantly fatal, or death may ensue in a few hours. In cases of an opposite character the symptoms will be less urgent, and the patient probably suffer no inconvenience, save what results from the temporary debility and faintness. The blood will soon coagulate, and by the pressure which it exerts upon the orifice of the bleeding vessel, a mechanical obstacle will be opposed to its further effusion.

When the quantity of fluid poured out is considerable a tumor is sometimes formed, which may be easily detected by its prominence and hard feel. If the patient survive the immediate shock of the accident, he may die from inflammation, caused by the clotted blood acting as an extraneous substance. At other times the coagula are absorbed, or they become encysted by an exudation of plastic lymph.

In the *diagnosis* of a wounded bowel important information may frequently be obtained, in regard to the direction, extent, and depth of the lesion, by a careful consideration of the shape and size of the vulnerating body. When the outer opening is so large as to admit the finger, it will generally be easy to determine whether the injury reaches the cavity of the abdomen: probing with instruments is quite inadmissible; it can do no good, and may occasion much harm. It need hardly be observed that it is highly proper, in every inquiry of this kind, to place the patient as nearly as possible into the posture in which he was at the moment of the accident. When the wounded bowel protrudes at the external opening, the diagnosis is at once obvious, as the nature and extent of the injury may be determined by simple inspection. The lesion, in the absence of pathognomonic symptoms, ought to be suspected when nausea and vomiting occur after penetrating wounds of the abdomen, accompanied with griping pains, great debility and faintness, jactitation, extreme anxiety, and cold sweats. The case is plain enough when there is a discharge of the contents of the alimentary tube, or a sudden development of tympanites.

It not unfrequently happens that an instrument enters the abdomen, and passes out at the opposite side, without, in the slightest degree, interfering with the bowels or other viscera. Many interesting cases of the kind are related by writers. I select the following in illustration of the subject.

A young soldier received, in a duel, a thrust from a sabre on the anterior part of the abdomen, a little above and to the right of the umbilicus. The walls of the belly were divided, and a considerable mass of omentum protruded through the opening. The patient was removed to the hospital, where every attempt was made to reduce the prolapsed parts, but without success. Blood was freely abstracted from the arm, leeches, cups, and fomentations were applied to the abdomen, and perfect quietude was enjoined; in short, every thing was done to prevent peritoneal inflammation. Eight days after the reception of the injury the extruded omentum was cut off, after which the wound became covered with healthy granulations, and at the end of fiveweeks the man was nearly well.*

The following case, mentioned by Sir Astley Cooper,† is strikingly illustrative of the manner in which the intestines glide away from the edge of the instrument. He was called to a female whom he found lying on the floor, weltering in her blood, from the infliction of four wounds upon her throat, in an attempt to commit suicide. Having closed these with sutures, his attention was directed, by some incoherent remark which she made, to her abdomen, where he found the bowels exposed by a wound reaching nearly from the pubes to the ensiform cartilage of the sternum. After cutting her throat with a razor, she had ripped up her belly with it, and let out her bowels, which were still distended with air, and had not sustained the slightest injury.

Dr. Hennen states‡ that he was witness to the recovery of

---

*Medico-Chir. Review, vol. ix, p. 527.

†Op. cit., vol. ix, p. 528.

‡Principles of Military Surgery, p. 319. Phila., 1830.

a soldier who was shot through the body with a ram-rod at the siege of Badajos, in 1812. The instrument entered the front of the abdomen, and actually stuck in one of the transverse processes of the vertebræ, from which it could not be disengaged without force. An analogous case is related by Dupuytren.*

A man in a fit of severe grief resolved to put an end to his existence, and for this purpose rushed with all his force against the point of a sword, which he had previously fastened in the wall of his apartment. So completely was the abdomen transfixed that the point of the weapon stuck out for eight or ten inches on the right side of the spine. When Dupuytren saw him, he seemed to suffer but little pain, and there was no symptom of any extravasation, or, indeed, of a wound of any of the abdominal viscera. It required considerable force to withdraw the sword. By repeated bleedings and the employment of a very rigid antiphlogistic regimen, the patient speedily recovered.

Richard Wiseman mentions the case of a young man who was run through with a rapier, which entered at the right hypochondriac region, and passed out at the back. On the the following day his skin was hot, and the pulse somewhat accelerated, but there was no tension of the abdomen, colic, vomiting, or any thing denoting injury of the intestine, or any other viscus, and he recovered in a very short time. "Thus," says Wisemen, "it frequently happeneth that a sword passeth through the body without wounding any considerable part.†" Two similar cases are recorded, one by Lamotte,‡ and the other by Garangeot, in each of which a sword passed directly across the cavity of the abdomen, without injuring a single fold of the intestinal tube.

Numerous instances of penetrating gunshot wounds of the

---

*Medico-Chir. Review, vol. xxi, p. 301.

†Chirurgical Treatises, 4to., p. 373. London, 1676;

‡Traite Complet de Chirurgie, T. ii.

abdomen are recorded, in which the bowels appear to have completely escaped injury. A case, which was evidently of this nature, is mentioned by Dr. John W. Richardson, of Tennessee, in the fourth volume of the Western Journal of Medicine and Surgery.* The ball, which weighed two drachms and a half, entered the abdomen on the right of the median line, and issued midway between the last rib and the sacro-iliac symphysis, immediately on the right side of the spine. There was no escape of gas or fæculent matter from the wound; some bloody urine was discharged soon after the infliction of the injury, and for the first eight or ten days there was considerable tension with soreness and swelling of the abdomen. The whole treatment was very simple, and the patient recovered in less than a month.

When the ball does not pass entirely through the body, it may be retained in the peritoneal cavity, or, if it wound the bowel, it may at once fall into the latter, and be discharged by stool. In the former case the foreign body excites adhesive inflammation, by which it becomes encysted; after it has remained in this condition, however, for a while it usually induces suppurative action, which gradually extends to the coats of the intestine, and finally produces perforation, whereby an outlet is established for its evacuation. When the extraneous substance is very small, as, for example, a shot, or even a small bullet, it occasionally continues encysted for many years, or even during the remainder of life, without occasioning any ill effects. An instance in which a number of encysted shot were found in the peritoneum recently occurred in the Louisville Marine Hospital, in an old man who had been wounded by a musket ten or twelve years previously. He soon recovered from the injury, to which he never referred any of his subsequent ailments.

I shall conclude this citation of authorities with the following extraordinary case recorded by Dr. Hennen, in his work

---

* This case is reported as having involved the colon and small intestines, without any evidence whatever that this was the fact.

on Military Surgery. A soldier of the Brunswick corps was wounded on the 16th of June, 1815, by a grape shot, which struck the right arm near the elbow, the articulation of which was destroyed. An English surgeon amputated the limb some hours after. The patient remained that night at Genappes. Next morning he observed blood flowing through the bandages, and requested Dr. Spangenberg, physician-in-chief to the Hanoverian army, to examine the arm. He found the humerus split as far as the joint, and with the consent of the patient immediately extracted it. After having dressed the parts, the man complained of pain in the abdomen, which was ascertained to proceed from a wound caused by a grape shot, which had passed through the exterior part of the belly, leaving two openings, one in front and the other behind, through each of which a portion of intestine protruded, not injured or inflamed, but in the natural state. The bowel, smeared with oil, was carefully reduced, and the two apertures were covered with adhesive plaster. The patient was brought to the hospital at Laecken, on the 19th of June, with moderate fever, and very little pain in the abdomen, or in the wound of the arm. The functions of the intestinal tube were not disturbed. He took little or no medicine; in four weeks the sores in the arm were cicatrized, and those of the abdomen, which were slightly affected with gangrene, in about three months.

The *prognosis* of wounds of the intestines must necessarily be influenced by a great variety of circumstances, such, particularly, as the extent of the mischief, the nature of the vulnerating body, and the state of the patient's health at the time of the accident. A small and simple lesion will be much more likely to turn out favorably than one involving a large surface, or one complicated with injury of some other organ, or the perforation of a large vessel. It is also less serious in an incised than in a contused or lacerated wound, and in a superficial than a deep one. Persons occasionally perish from the most trivial accidents of this kind, from the mere shock probably of the nervous system; they lie in a pale and exhausted condition, and death takes place unpreceded by reaction. On the other hand, recovery sometimes occurs under circumstances apparently the most desperate and unpromising. No certain rule can, therefore, be laid down in respect to the prognosis of wounds of this description; which, however, must always be considered as severe accidents, likely to be followed by the worst consequences. Wounds of the large bowel were regarded by the ancient surgeons as less serious than those of the small; a view in which most modern authors seem to concur. The reason of this difference is, first, the more fixed condition of the lower portion of the tube; secondly, its more capacious calibre; and thirdly, the more solid nature of its contents. These circumstances may all be supposed to be favorable to the prevention of the effusion of fæcal matter. Extravasation will also be less apt to occur when the bowel is empty than when distended.

When the contents of the bowel are effused over the peritoneum, death is sure to take place from the effects of inflammation. Occasionally, as was before intimated, life seems to be destroyed by the shock sustained by the nervous system within a few hours after the accident, and before the constitution has had time to rally. The fæcal extravasation, when slight, is sometimes limited by the deposition of plastic lymph, and the discharge of it is ultimately promoted by the formation of an abscess; or chronic action is established in the serous membrane, and the patient, after weeks or months of suffering, sinks under the exhausting influence of the malady. In the great majority of instances, however, death is induced by acute peritoneal inflammation. The symptoms presented are violent burning pain of the abdomen with great tenderness on pressure; intense thirst; a sharp, frequent, and contracted state of the pulse; constipation of the bowels; coldness of the extremities; constant wakefulness; great anxiety and restlessness. In the latter stages there is generally some degree of nausea with occasional vomiting; the pulse is weak and fluttering; the surface is bathed with a cold clammy sweat; the features are collapsed; the breathing is oppressed and laborious; the belly extremely tense and tumid; the patient is harassed with cough, his strength rapidly forsakes him, and he dies under all the symptoms of one sinking from the effects of mortification. The attack rarely continues beyond forty-eight hours, and often terminates fatally in a much shorter period. The appearances after death are always well-marked when the disease has been protracted. The peritoneal surface is highly inflamed, the bowels are covered with lymph, and the abdominal cavity usually contains a small quantity of turbid serum. The intestinal coils are frequently united to each other and to the neighboring parts, and on penetrating the belly there is almost always an escape of fœtid gas.

### IV.—*Mode of Reparation.*

I come, in the next place, to consider the process employed by nature in repairing wounds of the intestinal tube, and the mode in which she disposes of the ligature used in securing their edges.

If a small circular ligature be drawn firmly round the bowel of a dog, or other animal, the resulting effects will be very similar to those which attend the ligation of an artery. The opposite surfaces will not only be forced into close contact with each other, but it will produce at the same time a complete division of the mucous coat. If the cord be pulled very tightly, there will be in addition, especially in young subjects, a partial separation of the cellulo-fibrous lamella and of the muscular fibres. These effects I have repeatedly witnessed in my experiments on dogs, and they may be readily produced in the human body after death. If a flat ligature be used, even when it is drawn with considerable firmness, the opposite surfaces of the tube are merely brought into contact, without any rupture of the substance of any of the tunics. The only exception to this is where the animal is very young and the parietes of the bowel are unusually tender; in which case there will be occasionally a slight division of the lining membrane, but not of the muscular fibres. When a narrow ligature is used, the parts above and below it are so closely approximated that they touch in the greater portion of their circumference: a circumstance which must necessarily exert a most favorable influence over the reparative process and the re-establishment of the continuity of the canal.

Soon after an operation of this kind, in which a narrow circular ligature is used, inflammation is set up, plastic lymph is deposited upon and around the constricted parts, ulcerative absorption is established, and the cord at length works its way into the intestinal tube, where it is discharged along with the fæces. The period required for the detachment of the ligature may be supposed to be influenced by various circumstances, the principal of which are referable to the form and size of the foreign substance, together with the force with which it is applied, the thickness of the different tunics of the bowel, the age of the subject, and the state of the general health at the time of the operation, as well as immediately after it. In a small but full grown dog, killed at the end of the third day after the experiment, the ligature, which was round and narrow, had found its way through more than onehalf of the circumference of the tube, and in another animal of the same kind, which died from the effects of the operation thirteen hours later, the progress of the foreign body was still greater. In the latter, indeed, the cord had entirely disappeared, having lost its hold, and escaped into the bowel, in which, after a minute examination, it was discovered at the distance of several feet from the seat of the injury, surrounded by fæcal matter. In both cases the continuity of the parts was thoroughly re-established by an abundant deposition of lymph, which, notwithstanding the brief period that had elapsed, exhibited already well-marked traces of organization. The bowel, however, presented in each instance a constricted appearance; and in one of the animals, that, namely, which was killed at the end of the third day, the opposed mucous surfaces were still in close contact, no attempt having apparently been made to restore that portion of the tube. In the other the parts were not only perfectly continuous with each other, as has just been intimated, but the cavity was partially re-established. In a third experiment, performed on a middle-sized dog, not more than eighteen months old, the ligature was found lying at the seat of the constriction, where it was retained by a layer of plastic lymph, which had sealed up, as it were, the surface of the fissure in the mucous tunic. The canal of the bowel was completely restored, and the bond of connexion between the divided parts firm and organized. The animal was killed on the eleventh day.

The following experiment was performed by Mr. Travers, and is recorded in his work on wounds of the intestines. A ligature of thin pack thread was firmly tied around the duodenum of a dog, so as completely to obstruct it. The ends of the string were cut off, and the part returned. On the fifteenth day, his cure being established, he was killed. A portion of omentum connected to the duodenum was lying within the wound, and the folds contiguous to the strictured intestine adhered to it at several points. A slight circumferential depression was observed in the duodenum, and the mucous surface was more vascular, as well as of a deeper color, than usual. A transverse fissure marked the seat of the ligature. The edges of the sections were distinctly everted, and the appearance corresponded with that of the union by suture.

The lymph which is effused upon the external surface of the bowel, consequent upon the operation, gives the part at first a rough uneven appearance; but after a few weeks, sooner or later, according to circumstances, it undergoes a sort of modelling process, and hence, if the animal survive several months, it is generally no easy matter to determine the seat of the injury. In a dog which was killed four months after the experiment was performed, the reparation was so perfect that, had it not been for the attachment of a small process of omentum, it would have been impossible, by mere external inspection, to discover the place where the cord was originally applied, such were its smoothness and polish. Nor was this confined solely to the outer surface of the tube. Internally the cicatrization was almost as complete, the continuity of the mucous membrane having been every where re-established. There was scarcely even a seam at the original seat of the constriction.

It will thus be perceived that, from the rapid manner in which the ligature is detached, there is no danger that the animal will suffer much inconvenience from the want of a passage. Indeed, when the ligation is made in the small bowel, or high up in the large, the alvine discharge may go on with the same facility as before, making allowance of course for the pain which must necessarily attend an operation of such severity.

Effects similar to the above are produced when a ligature is applied round the edges of a small wound, that is to say, from two to three lines in diameter, provided it be drawn with sufficient firmness not to slip off. The cord gradually cuts through the different coats of the bowel, and the continuity of the canal is re-established by the effusion of plastic lymph upon the constricted part. The process of reparation, however, is not so speedily completed, owing to the breach being much wider than when a ligature is simply cast round the tube. In this case the mucous membrane is reproduced only after a long time, and the amount of lymph required is proportionally much greater. The ligature is detached at a period varying from five to ten days.

Wounds and punctures of the bowel, unaccompanied by the effusion of fæcal matter, heal, when left to themselves, either by the adhesion of their edges to the surrounding parts, or by the deposition of lymph upon their surface and the gradual approximation of their lips. In the majority of cases the reparation is probably effected by the former method; since there is always a great tendency in the wounded structures to attach themselves to those in their immediate vicinity. Even wounds of large size are occasionally repaired in this manner. In some instances, again, the breach is closed by a piece of omentum, which projects into it, and fills it up like a tampon. When this happens the serous membrane is firmly fixed to the edges of the opening, and the part which corresponds with the interior of the canal and assists in maintaining its continuity, is eventually absorbed; an occurrence which leads to the gradual approximation of the lips of the wound and their ultimate re-union. Jobert thinks that this mode of reparation is not uncommon, an opinion in which my observations do not induce me to concur. That it takes place occasionally is certain, for I have several times witnessed it in my experiments. He refers to a case, reported by Dr. Qurcial of Toulouse, of perforation of the jejunum, in which the epiploon projected into the opening, and thus effected a cure.* All the older surgeons, down to La Faye, Palfin,† and even Sabatier,‡ believed that wounds of the intestines never united, except through the intervention of the peritoneum, the omentum, or some of the neighboring viscera.

In mortification of the bowels, especially when occurring

---

* Traité des Maladies du Canal Intestinal, T. i, p. 66.

† Anatomie Chirurgicale, T. ii, p. 66.

‡ Medecine Operatoire, T. i, p. 33.

in small patches, the mode of reparation appears to be similar to that which takes place when a wound or puncture is left to itself. By the time the eschar is detached the edges of the breach will have formed adhesions to the circumjacent parts, by which the effusion of fæcal matter will be effectually guarded against. Where this is prevented the patient dies from peritoneal inflammation, or an artificial anus is established.

The subject of gunshot wounds of the intestines appears to have been more profoundly investigated by Baron Larrey than by any other surgeon. He divides the curative process into four stages. In the first, the bruised and lacerated tissues are deprived of their vitality, to an extent varying according to the amount of the injury they have sustained. In this respect a gunshot wound of the alimentary canal does not differ from that of any other part of the body. In the second stage, the eschar is detached, and the opening gives vent to fæculent and purulent matter, which continues to escape for several weeks or even months. During the third stage, the discharge gradually diminishes, and at last ceases altogether to appear externally. The union of the wound constitutes the fourth stage. The corresponding textures gradually approach each other, and, cicatrizing from within outwards, the whole chasm is at length completely filled up: the primitive adhesions become absorbed, and there only remains a slight contraction of the intestinal tube at the wounded part.*

When sutures are employed the mode of reparation is essentially alike, whatever may be their form. The inflammation which is lighted up induces an effusion of lymph, which is speedily followed by adhesion of the injured coil to the neighboring structures, among which it is sometimes completely burried. At other times no such adhesion occurs, but the affected part throughout the entire line of suture is coated

---

* Medico-Chirurg. Review, vol. xvi, p. 58.

with a layer of plastic matter, by which the continuity of the serous surface is finally re-established, and the threads used in sewing up the wound are concealed from view. In almost all cases—certainly in eight out of ten—there is an attachment of the omentum to the surface and edges of the wound, which thus assists, in an eminent degree, in the process of restoration. I speak now of course only of what I have noticed in dogs; whether the same thing takes place so readily, and to the same extent, in the human subject, my information does not enable me to determine. Probably it does not, as the epiploon is generally much smaller in man than in some of the inferior animals, especially in the canine races. The attachment of this membrane to the surface and edges of the wound is a very different matter, it will be observed, from the projection of it into the breach, in the manner pointed out and so strenuously insisted upon by Jobert. We have already seen that the latter is comparatively rare, while the other, on the contrary, is exceedingly frequent.

This extraordinary tendency to adhesion in the external surface and edges of the wound to the parts around it, is nothing more than what might be expected when we reflect upon the nature of the peritoneum, and its invariable disposition, when inflamed, to pour out lymph. But it is otherwise with the mucous membrane. Here the process of re-union is not only much slower but much less perfect; lymph is furnished very sparingly, or in quantities barely sufficient to fill the chasm between the margins of the wound; and, owing to the heterogeneous and irritating nature of the contents of the tube, a long time must necessarily elapse before it can become an organized or living intermedium. The little narrow band thus formed adheres firmly to the bottom of the wound, but very slightly, if at all, for some days, to its edges. Gradually, however, it becomes more and more dense; vessels extend into it from the circumjacent parts; its margins are flattened down; and, after a period varying from a few weeks to as many months, the adhesion is finally completed. Subsequently, or, indeed, while the changes just adverted to are still in progress, the new matter is nearly all absorbed, the wound greatly diminishes in width, and when the cicatrizing process is perfected merely a small depression or seam remains, to indicate the original seat of the injury. The whole process may be compared to that which nature employs in the reparation of ulcers of the mucous lining of the small and large bowel.

This, however, is only one mode in which the restoration of the mucous surface is effected. Another, though by no means a frequent one, is by granulation. It has been already stated that, owing to the irritating and heterogeneous character of the contents of the bowel, the lymph which is deposited upon the wound is very tardy in becoming organized, and it may now be added that this process is occasionally entirely prevented, the substance in question being either destroyed or removed by the fæcal matter as it passes over the affected part. When this happens, nature, faithful to her duties, makes an effort to repair the breach by the formation of granulations, as in similar injuries of other textures. The process under these circumstances is generally much more tardy than in the previous case, the cicatrization is also less complete, and the tube is much more apt to be puckered immediately around the seat of the injury. Mr. Travers seems to doubt that the fissure in the mucous lining is ever filled by granulation. "I had been led to expect," says he, "that the interstice of the villous coat would be filled by granulation, and that the substanee of the cylinder would in this way be restored at the place of division. But finding the eversion of the villous edges uniform and permanent, it seemed doubtful if such a process could be set up, as perfect surfaces were opposed to each other. It is also not inconsistent with the indisposition of mucous surface to the adhesive inflammation to infer that it does not readily admit of the granulating process, which is only an advanced stage of that inflammation."*

* Op. cit., p. 131.

I quote the language of this distinguished author, in order that his meaning may be fully understood. I am not aware that a similar opinion has been expressed by any other writer, and how so accurate an observer should have arrived at so erroneous a conclusion cannot be easily conceived. That granulations are formed on mucous surfaces is a matter of daily observation, and my researches have abundantly satisfied me that they are occasionally concerned in the restoration of the villous portion of a wounded bowel. The process of course is difficult; it must be so from the very nature of the mucous tissue, indisposed as it is to pour out plastic lymph; but this does not prove that it may not take place.

This writer has made another remark in relation to this subject not less erroneous, when he asserts that the adhesion which takes place between the mucous surfaces within a few hours after their connexion by suture is in no instance permanent, but that it is destroyed by the retraction of the divided parts when the ligatures drop off. Such an occurrence does undoubtedly sometimes take place, but I have repeatedly observed the reverse, and there is reason to believe, judging from the results of my own researches, that this happens much more frequently than is commonly supposed. Several days, often as many as eight or ten, must of necessity elapse before the sutures are detached; a period which is more than sufficient, in the plurality of cases, for the agglutination of the villous lips of the wound by plastic lymph. The apposition of the parts, moreover, is eminently favored by the crippled and paralysed condition of the muscular fibres at the seat of the injury, and by the tendency of the mucous membrane to eversion at the moment of the accident.

From the foregoing observations it is evident that the process of re-union is the same, whether the bowel be encircled partially or wholly by a ligature, whether we employ the suture, or, lastly, whether the wound, provided it be not too ample, be entirely intrusted to the resources of nature. In each case the restoration is effected through the medium of plastic lymph, poured out as a consequence of inflammation, and undergoing, sooner or later, a certain degree of organization.

The manner in which the ligatures are detached varies, as might be expected, according to the mode in which they are applied. Both in the interrupted and continued sutures, with their different modifications, the threads, provided their extremities are cut off close to the surface of the wound, invariably fall into the alimentary canal, along with the contents of which they are afterwards evacuated. This, indeed, may be laid down as an axiom, to which I saw no exceptions in any of my experiments, and which fully confirm, in this particular, as well as in many others, the researches of Smith, Thomson, Travers, and Cooper. The fact that the foreign body employed in making the suture is thus disposed of appears to have been first noticed, at all events hinted at, by Mr. Benjamin Bell in his System of Surgery; but it remained for two of the gentlemen whose names have just been cited, namely, Mr. Thomson and Mr. Travers, to settle the question by a direct appeal to experiments on the inferior animals.

The same circumstance, as was previously intimated, occurs when a ligature is cast around a loop of intestine, or when it is employed to encircle the margins of a small aperture, whether caused by injury or mortification. If, on the other hand, the extremities are permitted to hang out at the external wound, they will be discharged outwardly instead of inwardly, as in the former case. When the threads, through accident or negligence, slip beyond the reach of the operator, and escape into the peritoneal cavity, they will either induce fatal inflammation, or lymph will be poured out and they will thus become encysted, or they will excite ulcerative action in the coats of the bowel and find their way into it, or they will be evacuated through the opening in the wall of the abdomen.

### V.—*Treatment.*

Leaving this subject, I proceed to speak of the treatment of wounds of the intestines; a topic which necessarily involves the consideration of the suture in all its modifications and varieties.

In entering upon the discussion of this subject, for the elucidation of which my researches were mainly instituted, the first question that presents itself is, are there any circumstances in which the surgeon should feel himself justified in returning into the abdomen a wounded bowel without sewing it up, and, if so, what are they? This is a point, it must be conceded, of paramount importance, since it closely concerns not only the reputation of the practitioner, but, what is of much greater moment, the fate of the sufferer.

Penetrating wounds of the abdomen are not necessarily attended with protrusion of the bowels. Far from it. It is well-known that serious mischief is frequently inflicted, and yet, owing to the small size of the external opening, to the position of the body at the time of the accident, or to some other cause, there is not the slightest prolapse. In a case of this kind it does not matter, as a general proposition, what may be the extent or direction of the wound; whether, in other words, it be small or large, oblique, transverse, or longitudinal, since the treatment is to be conducted solely upon general principles, like that of any other internal or penetrating wound whatever. No probing is to be done, no dilatation practised, no suture employed. All that is required is to keep the patient quiet, and to resort to such means as are calculated to prevent inflammation, or, if this should arise, to limit its action. This is all that sound surgery demands, all that common sense indicates. Still, as there are no rules in grammar without exception, so there are very few, if any, in the healing art that do not admit of some deviation from established usages. This I believe to be eminently true in regard to the present subject. While, therefore, I would condemn as much as any one, and in language as emphatic as it is possible to express it, an indiscriminate recourse to the means just referred to, or not dilate the external wound and search for the injured bowel, with the view of sewing it up, simply because the patient had been hurt, I believe that circumstances may occasionally occur in which such a practice would not only be proper, but highly necessary to the safety of the individual. Let us, for the sake of being more fully understood, suppose a case: A man, after having indulged in a hearty repast, receives a penetrating wound in the abdomen from the thrust of a dirk or knife; the bowel is pierced, or, it may be, nearly divided, and there is a copious discharge of fæcal matter, both externally and into the peritoneal cavity, as is evinced, in the latter event, by the excruciating pain, the gastric oppression, and the collapsed condition of the sufferer. Here the most prompt and decisive measures must be resorted to, or the person will perish from peritoneal inflammation with as much certainty as if his skull had been fractured and a portion of his brain let out. It will not do for the surgeon to fold his arms, and look upon the scene as an idle and uninterested spectator. Far otherwise. He has a duty to perform, and that duty consists in dilating the external wound, if it be not already sufficiently large, in hooking up the injured bowel, and in closing the solution of continuity with the requisite number of stitches, at the same-time that the effused matter is carefully removed with tepid water and a soft sponge. All wiping must of course be studiously avoided, if it be possible to do it, as this would add much to the risk of peritonitis. After the bowel is exposed, and this should be done freely, if necessary, the water is to be pressed from the sponge so as to run over the affected surface in a full stream. This method, as I know from numerous trials, not only removes any foreign substance more easily than wiping, but is much less apt to be followed by unpleasant consequences.

By the above procedure, which, under the circumstances pointed out, I should never hesitate to pursue, the patient is not placed in a worse condition than a female who has undergone the Cæsarian section, or a person whose abdomen has been ripped up in the first instance; recovery from both of which is not, as is well-known, of unfrequent occurrence. A case in which a most extensive wound of the belly, with complete division of the ileum, and serious lesion of the thoracic cavity, was successfully treated by Mr. Calton, of Scotland, is reported in the twelfth volume of the Edinburg Medical and Surgical Journal, and another, in which still more frightful mischief was inflicted by a cannon-ball, and yet the man got well, is mentioned in Hennen's Military Surgery, and will be found in another part of this essay. A number of similar examples are scattered through the records of the profession, and could the experience of practitioners generally be ascertained in regard to this point it would be found, I doubt not, to afford a vast amount of additional evidence illustrative of this important subject. The truth is, the fatality of penetrating wounds of the abdomen has been greatly overrated. Injuries of this kind have been a sort of bugbear with surgeons and physicians, not so much from what they themselves have witnessed as from what they have heard from others; and hence a prejudice has arisen against the infliction of wounds and even punctures upon the peritoneum which has "grown with our growth and strengthened with our strength" until it has become almost impossible to eradicate it.

In making these remarks respecting the dilatation of the outer wound, for the purpose of enabling us to search for the injured bowel, let it be understood that I would recommend the practice only under particular circumstances. These circumstances have been already pointed out, and it is not necessary, therefore, to dwell upon them in this place.

When there is reason to suspect, from the nature of the vulnerating body, that the opening in the intestine is comparatively small, not exceeding, perhaps, the third or fourth of an inch in diameter, it would be extremely improper, if not absolutely unjustifiable, to search for the bowel with the view of sewing it up. Such a step, indeed, could not be too strongly reprobated, as it would seriously complicate an injury which, if left to itself, might easily heal.

The above remarks, with the reasoning founded on them, are fully borne out, if I mistake not, by some of the facts cited in a previous part of this inquiry, in relation to the escape of fæcal matter from the alimentary canal, when laid open to the extent of from four to six lines, whether longitudinally, transversely, or obliquely. In all cases of this kind, with scarcely a solitary exception, death is produced in from thirty-six to forty-eight hours by peritoneal inflammation. Mr. Travers, with many other respectable surgeons, is, I am aware, strongly opposed to the practice of dilating the abdominal wound and searching for the injured bowel, on the ground that the intestinal aperture retains its apposition with the parietal orifice; but he has adduced no experiments, or facts of any sort, in support of his conclusion, which is, besides, at variance with the existing state of our knowledge in relation to the subject. My own researches, at all events, have led me to a different result, and I can therefore see no just reason why the suggestion which I have ventured to throw out should not be adopted under the restrictions indicated.

The next topic into which I shall inquire is the conduct which the practitioner should observe when he is called to a penetrating wound of the abdomen, attended with protrusion, but no particular injury, of a portion of the alimentary canal. Cases of this description are by no means unfrequent, and they occasionally happen when the external opening is so small as to render it seemingly impossible for any prolapsion to take place. By the older surgeons such injuries were often treated in the most barbarous manner, and it is not improbable that serious harm is sometimes done by the ignorant and timid in our own day. Instead of reducing at once the extruded intestine, a procedure sanctioned both by theory and experience, a great deal of time used to be wasted in fomenting the part, in the vain hope that this would promote recovery; and when at length, by the delay thus occasioned, the gut became too painful to be replaced, instead of dilating the outer wound, they did not hesitate to leave it in its exposed situation; a practice which, as might have been supposed, was speedily followed by the death of the patient, or, what is scarcely less pitiable, an artifieial anus.

It is perfectly plain that in such a case the part should be at once restored, without the loss of a moment. It is certain that no good can be done by delay, while it is equally clear that it may be productive of much harm. Before the surgeon proceeds to the operation, the patient should be placed in the best possible position for relaxing the abdominal muscles. For this purpose he should lie on his back, the head being supported by a pillow, the pelvis elevated higher than the shoulders, and the lower extremities bent at the hips and knees. If the bladder be much distended, it should be previously emptied, and the patient should refrain from coughing, holding his breath, or any similar efforts calculated to impede the reduction. In a word, he should conduct himself precisely as if he were about to undergo an operation for strangulated hernia.

When these arrangements are effected, the surgeon, standing at the side of the patient that may be most convenient to him, takes the bowel into the left hand, while with the right he gently pushes it back, taking care to begin with the part which was protruded last, or which is nearest the wound. These efforts are to be continued until the whole slips into the abdominal cavity, when the external opening is to be closed in the manner to be pointed out presently, and the case treated upon general principles. Proceeding slowly and cautiously in this wise, the largest protrusions may generally be replaced without much difficulty, without inflicting any undue violence upon the patient, or without endangering the result by peritoneal inflammation. Nevertheless, it is sometimes almost impossible to effect the reduction, even when the prolapsion is inconsiderable, owing to the smallness of the external orifice, to the distended condition of the bowel, or to the spasmodic action of the muscular fibres, or to all these causes combined. Be this as it may, the best method under these circumstances is to enlarge the wound to the requisite extent by means of a probe-pointed bistoury, cautiously insinuated between the gut and the resisting parts. Some of the older surgeons, as Paré, Low, and Garangeot, were in the habit, when the difficulty depended upon inflation, or gaseous distention, of making punctures in the bowel to evacuate the contained air; a practice which was afterwards embraced by Gooch, Sharp, Sabatier, Chopart and Desault. The plan, as originally suggested, consisted in making the punctures with a small needle, which was replaced by a large round one in the hands of Chopart and Desault, who have described the operation with much minuteness. The procedure, however, was pointedly condemned by Blancard and La Faye, on the very sufficient ground of its inefficacy, as well as danger, and is now scarcely ever thought of, except as a matter of scientific curiosity. Others have recommended the substitution of a small trocar, but the same objections lie against it as against the use of the needle.

In our attempts to restore the bowel to the abdomen, it is all important to know that it has actually slipped into its natural situation. The route which the wound follows is occasionally very devious, or it may happen that there is a slight detachment of the peritoneum round the edges of the inner orifice, produced either in the first instance, or by the finger of the surgeon in his efforts at reduction. In either case, a most serious error may be committed by supposing that the protruded parts have been returned, when in reality they are retained on the outside of the serous cavity, where they may become strangulated, or affected with undue, if not fatal, inflammation. The operator should therefore never rest satisfied that the restoration has been accomplished unless he is convinced that the finger has been fairly within the abdomen, or in contact with the convoluted surface of the bowel.

Penetrating wounds of the abdomen are rarely unattended with some protrusion of the omentum. From the situation of this serous lamella, and from the manner in which it is spread over the surface of the bowel, it is indeed usually forced out first, and not unfrequently it is the only part prolapsed. However this may be, it should always be carefully returned, otherwise the greatest mischief is to be apprehended. A distinguished surgeon, Baron Larrey, has, it is true, advised us to let it alone, that is, neither to return it, nor to remove it by the knife or ligature; a practice recommended by some very eminent authorities. Soon after the accident, he observes, the extruded membrane swells, becomes thick and red, and assumes a rough, granulated aspect. These symptoms increase until the third day, after which they remain stationary for nearly a fortnight, when the part begins to shrivel, and is ultimately reduced without any operation.* Very few practitioners will, I presume, be disposed to follow this advice, which is, to say the least of it, singularly at variance with that of the best writers on penetrating wounds of the abdomen and the management of ruptures. That practice is undoubtedly the safest which most readily promotes the recovery of the patient, and that this desirable end is more promptly and perfectly attained by returning the whole of the prolapsed omentum at once into the abdomen, than by allowing it to remain in the situation pointed out by the Baron, no one can doubt. Both experience and common sense are in favor of the course of treatment so long pursued by the ablest surgeons, and I can therefore see no necessity for adopting a new one, especially when that method is of an equivocal character. It is a good maxim in surgery, as it is in morals, to let well enough alone.

It need hardly be remarked that, when the protruded parts are covered with dirt, fæces and blood, or other extraneous matter, they should be carefully cleansed before any attempt be made to restore them to their natural situation. The importance of this practice is too obvious to require any comment. The best article for this purpose is tepid water, either

---

* Medico-Chir. Review, vol. ii, p. 261. 1821.

alone or mixed with milk, applied by means of a sponge held some distance off. The stream thus produced is well calculated to detach the foreign substances, whatever they may be, without inducing any additional irritation. In no case should the parts be sponged or wiped, for reasons which it is unnecessary to specify. If the extraneous matter adhere with much firmness, it may be picked off with a pair of forceps, or some other instrument, and on no account should the bowel be replaced until it has been thoroughly cleansed.

Fomenting the extruded parts with infusion of chamomile flowers, oil, hops, or wine and water, as recommended and practised by the late Baron Larrey, can do no good, and ought to be avoided. The advice of the French surgeon, indeed, is decidedly objectionable, if not reprehensible. The abdominal organs are the best fomentors, and the sooner the protruded parts are brought into contact with them the better.

The omentum, when prolapsed along with the bowel, should always be reduced last, and care taken to spread it out as much as possible over the parts which it naturally covers. This can generally be easily done by means of the index-finger of the right hand introduced into the peritoneal cavity, and is calculated to prevent its subsequent protrusion between the edges of the wound; a circumstance which almost constantly happens when this precaution is neglected.

In regard to the management of the external wound, it is obvious that it must be conducted upon the same general principles as that of a solution of continuity in any other situation. Sutures should never be employed, except where they are imperiously indicated. It should be remembered that they are foreign bodies, which can never be resorted to without an increase of pain, or without endangering the development of too much morbid action. It is well-known, too, that when introduced into tendinous structures they are apt to excite a bad form of inflammation, and that, if inserted into muscular parts, spasm and even convulsions may be the consequence. Nevertheless, cases often do occur in which we cannot dispense with them. The wound may be unusually large, or the patient so restless and unmanageable as to render it impossible to prevent a recurrence of the protrusion unless the parts be sewed up. Under circumstances such as these we would not only be warranted in employing the suture, but we should be justly culpable if we neglected it. Dogs bear this treatment with perfect impunity, and many cases are recorded in which it was advantageously employed in the human subject. In making a suture in this situation the needle should be carried through the lips of the wound within a line and a half or two lines of the peritoneum, and the requisite number of threads placed before any of them are tied, in order to avoid injury to the omentum. The ends are then cut off, and the approximation perfected by means of adhesive strips, the whole being secured by a compress and broad bandage carried two or three times round the abdomen. At the expiration of thirty-six or forty-eight hours the ligatures should be cut away, as the parts will have sufficiently united to render them unnecessary. When the wound is very extensive some surgeons prefer the quilled suture, as it is termed, but for this there can seldom be any necessity, when the case is managed in the manner just mentioned.

Penetrating wounds of the abdomen, attended with lesion of the intestinal tube, constitute a class of injuries of a much more serious character than such as are accompanied merely by prolapse. The symptoms are generally more severe, there is more danger of peritoneal inflammation, and the treatment, especially when the opening is extensive, is altogether different; or, to speak more intelligibly, two wounds, involving different structures, exist, and consequently require different modes of management.

When the inner wound is large the treatment to be employed is sufficiently obvious, for no well educated surgeon would hesitate to resort at once to the suture, or to some other contrivance calculated to prevent fæcal effusion. It is only where the opening is small that doubts seem to be entertained respecting the proper course to be pursued. The question can only be decided by an appeal, not to the speculative views of professional men, but to direct experiment upon the inferior animals and observation upon the human subject. The evidence which I shall adduce will go far, if I mistake not, to settle this important point of pathology and practice.

Heister, who was confessedly one of the ablest anatomists and surgeons of his day, expressly states that all wounds of the intestines not exceeding the diameter of a goose-quill should be returned without stitching, which he asserts to be generally productive of severe pain, inflammation, and other bad symptoms.* Dionis says if the opening is very small, as for example, when it is made by a bodkin or pen-knife, it is not necessary to sew it up; nature, seconded by a rigid diet, being fully competent to effect a cure.† To the same import very much is the testimony of Palfin,‡ and of Sabatier. The former of these authors observes that whenever the opening is diminutive it is not necessary to sew it up, but simply to return the part, and to restrict the patient to the smallest possible allowance of food, barely sufficient to prevent starvation. "If the wound," says Sabatier, "is very slight, as when only a few muscular fibres are involved, it is needless to resort to the suture, since a cure may be accomplished without it." Sharp, in his Operations of Surgery,§ uses very nearly the same language. The opinion of Jobert, whose writings have been already several times quoted, is, that the wounded intestine may be safely returned, provided the opening does not exceed three lines. Where it is more extensive, as for instance half an inch, although reparation might possibly take place through the intervention of the epiploon, still there would be great danger of fæcal effusion,

---

* Travers, op. cit., p. 172.

† A Course of Operations, p. 53. English Edition, London, 1733.

‡ Anatomie Chirurg., T. ii, p. 76.

§ P. 9. London, 1784.

and hence he very justly concludes that it would be much better to sew it up.*

Richerand, also a modern writer, recommends a very different practice when the wound is very small, or does not exceed two or three lines.† His plan is to pass a loop of waxed thread through the mesentery, and to keep the inner wound as nearly as possible in apposition with the outer. The object is to afford a ready outlet to the fæcal matter, by the artificial anus which is thus established. This method, to which I shall hereafter recur, is not new with Richerand, but originated long ago with La Peyronie, an old French surgeon. Boyer remarks‡ that when the wound is more than four lines in extent enteroraphy becomes indispensable.

In a preceding part of this essay—page 8—several experiments are related which have a direct bearing on this subject. The particulars, however, it is not necessary to reproduce in this place. It will be sufficient to say that in the three experiments in which the wound did not exceed four lines, or the third of an inch, the animals promptly recovered, while in the remainder, five in number, and in which the opening was of greater extent, they all died of fæcal effusion. So far, then as these researches go, they tend to confirm the opinion of Heister, Sharp, Garangeot, and others, that a protruded bowel, in which there is only a very small wound, may be safely returned into the abdomen, without any apprehension of the escape of alvine matter. But would the surgeon be really justified in pursuing such a practice? I unhesitatingly aver that he would not, for the reason that, although this course may, in the generality of cases, be attended with success, yet it is liable to occasional failure, and should therefore be discountenanced. The introduction of a suture, which is all that can

---

* Maladies du Canal Intestinal, T. i, p. 72.

† Nosographie et Thérapeutique Chirurg., T. iii, p. 319. Paris, 1821.

‡ Traité des Maladies Chirurgicales, T. vii, p. 377. Paris, 1831.

be needed in a small wound, will assuredly add little either to the present suffering of the patient or to the danger of peritoneal inflammation; the operation is neither painful nor tedious, and, what is of far more consequence, always, when well performed, protects the individual from fæcal effusion. In several of my experiments death was produced, not from any undue injury inflicted upon the bowel from stitching or any rough manipulation, but from the interval between the sutures being so great as to prevent the perfect closure of the wound; a fact which should never be lost sight of in the management of a lesion of this kind. Whenever the contact is incomplete, the mucous membrane becomes everted. and interferes with the adhesive process. The more accurately this is obviated the less risk will there be of the escape of fæculent and other matter, calculated to induce fatal peritonitis. I do not care, therefore, how small the wound may be, if it is only a line and a half, or two lines in extent, it should by all means be sewed up. In this practice alone can there be perfect security for the patient. The villous membrane may, it is true, effect a temporary closure of the wound, but there is always danger that before adhesion can take place, the part will become so much relaxed as to lead to mischief.

In closing this branch of the present inquiry I cannot omit quoting the sentiments of an old and distinguished surgeon, whose works, highly popular in their day, have been too much neglected by modern practitioners. I allude to Mr. Benjamin Bell.* "However small," says he, "a wound of the intestine may be, it ought always to be secured with a ligature; for although it is alleged by some that we should rather trust to nature for the cure of a small opening than to insert a ligature, to me it appears that the opinion is by no means well-founded; insomuch that I would not leave even the smallest opening that could admit either fæces or chyle to

---

* A System of Surgery, vol. v, p. 281.

pass, without stitching it up. Much danger may ensue from omitting it; and the hazard of the patient cannot be increased by the practice being adopted."

Co-incident with this opinion of Mr. Bell is that of Mr. Lawrence, of London, whose views upon the subject are entitled to great weight, from the unusual opportunities which he has enjoyed for treating strangulated hernia. Adverting to the practice recommended by Jobert, and referred to in a previous paragraph, of replacing the bowel without suture, when the wound does not exceed three lines, he affirms that such a procedure would not only be hazardous, but unwarrantable in the present state of the science. "In case of such an opening in the intestine," says he, "I should employ suture; not considering it safe to return the bowel into the abdomen without this precaution."*

It might be supposed that, in a treatise professedly devoted to the subject, considerable space would be alloted to the therapeutic treatment of wounds of the intestinal canal. Such a course would undoubtedly be highly proper, if not, indeed, indispensable, if these lesions involved any thing peculiar in this respect; but when it is remembered that they are to be managed upon the same principles as wounds in other parts of the body, much discussion of this kind would, to say the least of it, be irrelevant.

After the bowel has been restored to its natural situation, whether enteroraphy has been employed or not, the first and most important object is to guard against the occurrence of peritoneal inflammation, as it is upon this that the safety of the case mainly depends. Perfect quietude in the recumbent posture, the early and copious abstraction of blood, especially if the patient be plethoric, or the wound extensive, and the most rigid observance of the antiphlogistic regimen, are the means upon which our reliance is to be placed in the first instance. If the bowels be not evacuated spontaneously in

* Treatise on Ruptures, p 301. London, 1838.

six or eight hours after the parts have been returned, a stimulating enema should be thrown into the rectum, but under no circumstances should the alimentary canal be disturbed by the administration of purgative medicines by the mouth, as these, however mild, will be likely to cause griping pains and to interfere with the reparative process. This plan is to be persisted in for at least three or four days, when a dose of castor oil may be given, or, which would be better, an ounce of sulphate of magnesia or soda. The more fluid the alvine matters can be rendered the less likely will they be to be arrested at the affected part, to derange the sutures, or to disturb the healing process. All drastic articles must be sedulously avoided, on account of their tendency to create gastric irritation, and to excite undue peristaltic action of the bowels; two circumstances concerning which we cannot be two much on our guard.

The pulse should be attentively watched, and as soon as re-action is fully established, blood must be taken from the arm by a large orifice, and while the patient is in the semi-erect posture. The amount to be abstracted must vary according to the indications of the case, particularly the age and constitution of the individual, the return, continuance, or increase of the local pain, the force and frequency of the pulse, and the extent of the injury. The first bleeding ought, in general, to be tolerably copious, but after this eight or ten ounces at each repetition will be sufficient. In this way we prevent inflammatory action, or moderate it, where it has already taken place, without inducing too much prostration. It should be recollected that the pulse in peritonitis is hard, wiry, and contracted, and that the practitioner, if he be not fully aware of this, will be apt to fall into the error of omitting the abstraction of blood at a period when it is loudly called for, and when it can alone be of any avail in arresting the progress of the malady. General bleeding, however, is not always admissible. The shock which the system has received may be unusually severe; the reaction may be tardy and imperfect; and the patient may perhaps be for several days in a dosing state, with a weak and tremulous pulse, cold extremities, and great pallor of the countenance. In such a case, instead of taking blood from the arm, the practitioner must content himself with fomentations to the abdomen, consisting simply of warm water, or of water in which hops, opium, or poppy-heads have been infused, and frequently renewed. Even leeches are scarcely to be thought of. Where the stomach is irritable, mustard poultices are to be applied to the epigastric region, and if the patient is unable, as he occasionally is, to void his urine, it must be drawn off with the catheter. If cough be present, it is to be combated by the usual means, and not allowed to progress, as the concussion thus induced might prove highly detrimental. When the patient is harassed with colicky pains, relief may be attempted by laudanum or the salts of morphia, but as the effect of these and similar articles is to create constipation, they should be employed as sparingly as possible. The tenesmus which is sometimes present is to be allayed by anodyne injections or suppositories; and where there is much discharge of blood from the bowels, the acetate of lead may be administered in large and repeated doses.

When there is much tumefaction of the abdomen with gastric irritability, and tenderness on pressure, Baron Larrey* advises cupping, aided by camphorated and oily embrocations, emollient cataplasms, and anodyne enemeta. In a case, apparently of the most hopeless character, in which this practice was put in force, the disease yielded in a very short time, not, however, without vesication of the whole surface of the abdomen. With cupping I have no experience in the treatment of peritoneal inflammation, traumatic, or otherwise; but it seems to me that it would be attended with so much suffering to the patient as to preclude its employment in

---

* Surgical Essays, translated by Dr. Revere, p. 235.

most, if not all cases of the kind. Leeching would certainly be preferable.

The diet must be of the most simple nature. For the first fortnight or three weeks, it should consist chiefly of amylaceous articles, as arrow root, tapioca or sago; afterwards it may be more nutritious, but must still be fluid. Solid, stimulating, or flatulent food is not to be used for several months after the accident. Two or three cases will hereafter be mentioned, where, from disregard of this precaution, the patient fell a victim to his imprudence, when he was apparently out of all danger. As a constant drink, nothing can be better than cold water, flax-seed tea, slippery-elm water, or a solution of gum-arabic, simple or acidulated. In a word, the patient should be half-starved, and as much depleted as is consistent with the restorative process. Our treatment must be prompt and energetic. No time is to be lost, or the case will slip out of our hands. The great error with most practitioners is that they do not abstract blood sufficiently early, or before peritoneal inflammation is thoroughly established, or has made such inroads upon the system as to render it impossible to arrest its progress.

When blood is extravasated in considerable quantity into the peritoneal sac, as is evinced by the soft and tremulous state of the pulse, the pallor of the countenance, the coldness of the extremities, and the constant disposition to swooning, the patient must be immediately placed in the recumbent posture, and made to take large and frequently repeated doses of the acetate of lead in union with opium. Mustard poultices should be applied to the hands and feet, and cloths, wrung out of cold water, to the abdomen, which is to be encircled at the same time with a broad bandage, to afford equable support to the viscera, and thereby promote the coagulation of the effused fluid. When there is reason to suspect that a large artery has been opened, the most effectual practice will be to cut down upon the parts, and secure it with a ligature. This procedure, however, has few advocates, and should only be employed as a dernier resort, not as a matter of choice. It would certainly be better to make an effort to save the patient by an operation, even of a desperate character, than to allow him to perish from the loss of blood, when the wounded vessel is within our reach.

The dressings must be light, simple, and unirritating. If there be a discharge of fæculent matter, as there may be when the internal wound has not been properly sewed up, or even where there has been no protrusion in the first instance, it should be disturbed as little as possible, until there is reason to believe that the bowel has contracted firm adhesions to the surrounding parts. By disregarding this precaution fatal effects might ensue from the extravasation of the matter into the peritoneal cavity. During the whole treatment the utmost attention should be paid to cleanliness. As the external opening diminishes, means are to be employed to prevent the escape of fæces, by which the patient will be rendered more comfortable, and the healing process expedited.

When the patient is well enough to sit up or walk about, the weakened parts should be supported by a compress and broad bandage, or, what is better, a good truss, which should be worn day and night, to prevent the separation of the edges of the sore, and the protrusion of the contents of the abdomen. This caution, as has been justly observed by Mr. Benjamin Bell, ought to be persisted in for a considerable time after the cure has been completed. By a want of attention to this point, very troublesome cases of hernia have occurred, which might otherwise have been obviated.

Patients who have recovered from wounds of this kind must pay particular attention to their bowels, which should be kept in a soluble condition, and on no account be allowed to be costive, even for a single day. They should also be extremely temperate in their diet, and carefully masticate their food before it is swallowed. All rough exercise, as riding on horse-back, jumping, running, and even rapid walking, must be avoided.

(*To be continued.*)

## Figures and Tables

**Figure f1:**
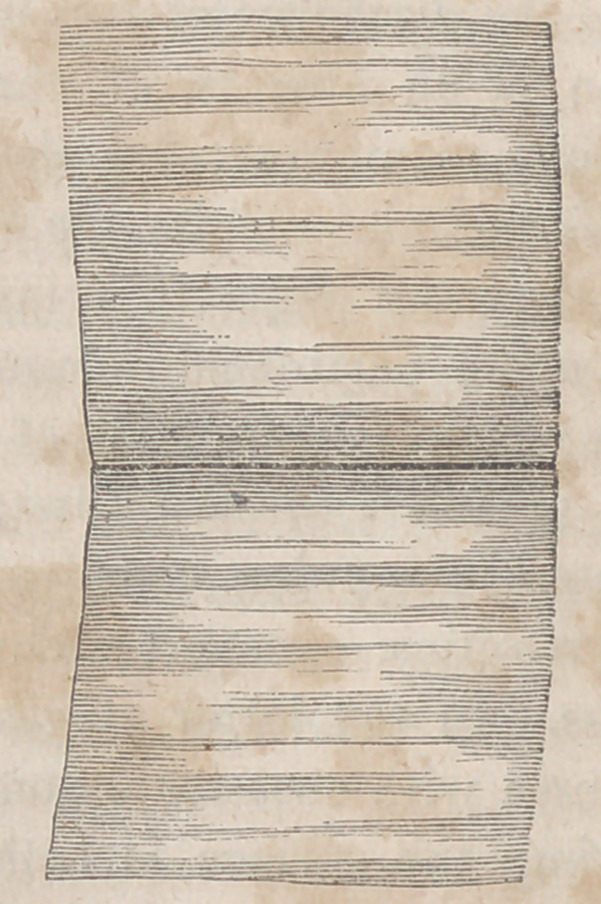


**Figure f2:**